# Supplementary material for: Identification of the EdcR Estrogen-Dependent Repressor in Caenibius tardaugens NBRC 16725: Construction of a Cellular Estradiol Biosensor
Source: Genes (Basel). 2021 Nov 23;12(12):1846. doi: 10.3390/genes12121846 (PMC8700777; doi:10.3390/genes12121846)
Supplement: Supplementary file 1 [file genes-12-01846-s001.zip › genes-1456427-supplementary.pdf]

Table S1. Primers used in this study. Restriction enzymes used for cloning are shown in brackets.

Primers	Sequence (5'-3')	Use
5BamHIREG1UPf	CATACGGATCCTCGAGCTTGCGGAAATAGGG	*EGO55_13520* deletion (amplification of upstream region) (*Bam*HI/*Sal*I)
3SalIREG1UPr	CACAGTCGACATGGGTTAGGCAGGGCATC	
5SalIREG1DOWNf	CACAGTCGACTTGTGATGGTGCGCGTGATC	*EGO55_13520* deletion (amplification of upstream region) (*Sal*I/*Hin*dIII)
3HindIREG1DOWNr	ACATAAGCTTCGAACTGATGCACCTGCTGA	
F24	CGCCAGGGTTTTCCCAGTCACGAC	Internal primer: used to check insert cloning in pK18mobsacB and to check insertion of pK18mobsacB
R24	AGCGGATAACAATTTCACACAGGA	
extREGf	TCGATAAAGGCCTTGGCGAA	External primer. Together with F24 used to check insertion of pK18edcR. Together with extREGr used to check *EGO55_13520* deletion
extREGr	TGATCCTCAACCGTGTTCCG	External primer. Together with R24 used to check insertion of pK18edcR. Together with extREGf used to check *EGO55_13520* deletion
T0pSEVA237F	CCGAGCGTTCTGAACAAATCC	Internal primer: Together with R24, used to check insert cloning in pSEVA237*PlexA*. Hybridizes in T0 terminator
3SEQIntRegGFPr	ACATTTAATTAAACTCCAGTGAAAAGTTCTTCTCCT	Internal primer: Together with R24, used to check insert cloning in pSEVA237*PlexA*. Hybridizes in *gfp* gene
5BamHICOMPReg2f	ATACGGATCCAGGAGGAAACATTTGGCGAATGAACTGAAGGCTG	Cloning *EGO55_13520* into pSEVA237*PlexA* (*Bam*HI/*Spe*I)
3SpeICOMPReg2r	CACATACTAGTTCAGGCCGCGCTGGAA	
5EcoRIStopIntRegF	ACATGAATTCCTAGGCAGCACCATCCGCCC	Cloning P_a promoter into pSEVA237*PlexA* (*Eco*RI/*Sal*I)
3SalIIntRegR	ACATGTCGACGGTTGCGCGCCGGAGT	
5EcoRIStopIntReg1f	ATATCGAATTCCTAATCGTCGTAGATGGCGGAAAC	Cloning P_b promoter into pSEVA237*PlexA* (*Eco*RI/*Sal*I)
3SalIIntReg1r	ACATGTCGACAAAAAACGCACTTTCGATACTATTGGT	
5PacIPlexAtetRT0f	ACATTTAATTAAAGCGGATAACAATTTCACACAG	Cloning *EGO55_13520* gen together with P_{lexA} promoter form pSEVA23edcR into pSEVA651 (*Pac*I/*Spe*I)
3SpeICOMPReg2r	CACATACTAGTTCAGGCCGCGCTGGAA	
5RTRecAf	CGATCGACGTGCTGGTGATC	RT-PCR, *EGO55_01665* (*recA*) amplification
3RTRecAr	TGCGGACCTGGTTGATGAAA	
5RTCoASynf	CCTGCTTCTGGCTGACGATG	RT-PCR, *EGO55_13555* amplification
3RTCoASynr	TGTCGACGATTTCCTCTGCA	
5RTTonBf	ACTCGTCCGATACCGTTTCG	RT-PCR, *EGO55_13600* (*edcT*) amplification
3RTTonBr	TGTGTTGCGACCATAGAGCG	
5RTDioxf	CGCTATCTTGGGCTGGAGG	RT-PCR, *EGO55_13570* (*edcB*) amplification
3RTDioxr	CGATATCCGGTCGTCACCAG	

Figure S1. Nucleotide sequence of pSEVA237M*Pb*. Hybridation sequences of oligonucleotides used to clone P_b into pSEV237 backbone are underlined. Scars left after ligation of P_b promoter fragment into pSEVA237M-BCD2 backbone are shown in bold. The synthetic bicistronic RBS, BCD2, is shown in italics.

TTAATTAAAGCGGATAACAATTTCACACAGGAGGCCGCCTAGGCTAATCGTCGTAGATGGCGGAAACCGCGGCGGGATTCT
GTTCGCATGCCCTGACAGCGGAAGTAGGAAAGCGGTCGTCGCATAACGCCCGCACGGAGCGCTGCAGCGCGTCCTGCTCTT
CCGATATGCTGAGGTCCATCCCGCTCTTCTCCCATCCGGTTGCGCGCCGGAGTTGCCATTAACGAAACTGTCGGTACCTAT
AAAATTTCTGTCTGTCCAGCCCTGTGTTCATGGCACAATCTGCTGGCCGGGTCTCTTGACTCCTAATAACAGTACCAATAG
TATCGAAAGTGCGTTTTTTGGATCC**TCTAGG***GCCCAAGTTCACTTAAAAAGGAGATCAACAATGAAAGCAATTTTCGTACT*
*GAAACATCTTAATCATGCTAAGGAGGT*TTTCTAATGATCATGGGAATTCATAAAGGTGAAGAACTGTTCACCGGTGTTGTT
CCGATCCTGGTTGAACTGGATGGTGATGTTAACGGCCACAAATTCTCTGTTCGTGGTGAAGGTGAAGGTGATGCAACCAAC
GGTAAACTGACCCTGAAATTCATCTGCACTACCGGTAAACTGCCGGTTCCATGGCCGACTCTGGTGACTACCCTGACCTAT
GGTGTTCAGTGTTTTTCTCGTTACCCGGATCACATGAAGCAGCATGATTTCTTCAAATCTGCAATGCCGGAAGGTTATGTA
CAGGAGCGCACCATTTCTTTCAAAGACGATGGCACCTACAAAACCCGTGCAGAGGTTAAATTTGAAGGTGATACTCTGGTG
AACCGTATTGAACTGAAAGGCATTGATTTCAAAGAGGACGGCAACATCCTGGGCCACAAACTGGAATATAACTTCAACTCC
CATAACGTTTACATCACCGCAGACAAACAGAAGAACGGTATCAAAGCTAACTTCAAAATTCGCCATAACGTTGAAGACGGT
AGCGTACAGCTGGCGGACCACTACCAGCAGAACACTCCGATCGGTGATGGTCCGGTTCTGCTGCCGGATAACCACTACCTG
TCCACCCAGTCTAAACTGTCCAAAGACCCGAACGAAAAGCGCGACCACATGGTGCTGCTGGAGTTCGTTACTGCAGCAGGT
ATCACGCACGGCATGGATGAACTCTACAAATAAGAATTCGAGCTCGGTACCCGGGGATCCTCTAGAGTCGACCTGCAGGCA
TGCAAGCTTGCGGCCGCGTCGTGACTGGGAAAACCCTGGCGACTAGTCTTGGACTCCTGTTGATAGATCCAGTAATGACCT
CAGAACTCCATCTGGATTTGTTCAGAACGCTCGGTTGCCGCCGGGCGTTTTTTATTGGTGAGAATCCAGGGGTCCCCAATA
ATTACGATTTAAATTTGTGTCTCAAAATCTCTGATGTTACATTGCACAAGATAAAAATATATCATCATGAACAATAAAACT
GTCTGCTTACATAAACAGTAATACAAGGGGTGTTATGAGCCATATTCAGCGTGAAACGAGCTGTAGCCGTCCGCGTCTGAA
CAGCAACATGGATGCGGATCTGTATGGCTATAAATGGGCGCGTGATAACGTGGGTCAGAGCGGCGCGACCATTTATCGTCT
GTATGGCAAACCGGATGCGCCGGAACTGTTTCTGAAACATGGCAAAGGCAGCGTGGCGAACGATGTGACCGATGAAATGGT
GCGTCTGAACTGGCTGACCGAATTTATGCCGCTGCCGACCATTAAACATTTTATTCGCACCCCGGATGATGCGTGGCTGCT
GACCACCGCGATTCCGGGCAAAACCGCGTTTCAGGTGCTGGAAGAATATCCGGATAGCGGCGAAAACATTGTGGATGCGCT
GGCCGTGTTTCTGCGTCGTCTGCATAGCATTCCGGTGTGCAACTGCCCGTTTAACAGCGATCGTGTGTTTCGTCTGGCCCA
GGCGCAGAGCCGTATGAACAACGGCCTGGTGGATGCGAGCGATTTTGATGATGAACGTAACGGCTGGCCGGTGGAACAGGT
GTGGAAAGAAATGCATAAACTGCTGCCGTTTAGCCCGGATAGCGTGGTGACCCACGGCGATTTTAGCCTGGATAACCTGAT
TTTCGATGAAGGCAAACTGATTGGCTGCATTGATGTGGGCCGTGTGGGCATTGCGGATCGTTATCAGGATCTGGCCATTCT
GTGGAACTGCCTGGGCGAATTTAGCCCGAGCCTGCAAAAACGTCTGTTTCAGAAATATGGCATTGATAATCCGGATATGAA
CAAACTGCAATTTCATCTGATGCTGGATGAATTTTTCTAATAATTAATTGGACCGCGGTCCGCGCGTTGTCCTTTTCCGCT
GCATAACCCTGCTTCGGGGTCATTATAGCGATTTTTTCGGTATATCCATCCTTTTTCGCACGATATACAGGATTTTGCCAA
AGGGTTCGTGTAGACTTTCCTTGGTGTATCCAACGGCGTCAGCCGGGCAGGATAGGTGAAGTAGGCCCACCCGCGAGCGGG
TGTTCCTTCTTCACTGTCCCTTATTCGCACCTGGCGGTGCTCAACGGGAATCCTGCTCTGCGAGGCTGGCCGTAGGCCGGC
CCTACCGGCGCGGCAGCGTTACCCGTGTCGGCGGCTCCAACGGCTCGCCATCGTCCAGAAAACACGGCTCATCGGGCATCG
GCAGGCGCTGCTGCCCGCGCCGTTCCCATTCCTCCGTTTCGGTCAAGGCTGGCAGGTCTGGTTCCATGCCCGGAATGCCGG
GCTGGCTGGGCGGCTCCTCGCCGGGGCCGGTCGGTAGTTGCTGCTCGCCCGGATACAGGGTCGGGATGCGGCGCAGGTCGC
CATGCCCCAACAGCGATTCGTCCTGGTCGTCGTGATCAACCACCACGGCGGCACTGAACACCGACAGGCGCAACTGGTCGC
GGGGCTGGCCCCACGCCACGCGGTCATTGACCACGTAGGCCGACACGGTGCCGGGGCCGTTGAGCTTCACGACGGAGATCC
AGCGCTCGGCCACCAAGTCCTTGACTGCGTATTGGACCGTCCGCAAAGAACGTCCGATGAGCTTGGAAAGTGTCTTCTGGC
TGACCACCACGGCGTTCTGGTGGCCCATCTGCGCCACGAGGTGATGCAGCAGCATTGCCGCCGTGGGTTTCCTCGCAATAA
GCCCGGCCCACGCCTCATGCGCTTTGCGTTCCGTTTGCACCCAGTGACCGGGCTTGTTCTTGGCTTGAATGCCGATTTCTC
TGGACTGCGTGGCCATGCTTATCTCCATGCGGTAGGGGTGCCGCACGGTTGCGGCACCATGCGCAATCAGCTGCAACTTTT
CGGCAGCGCGACAACAATTATGCGTTGCGTAAAAGTGGCAGTCAATTACAGATTTTCTTTAACCTACGCAATGAGCTATTG
CGGGGGGTGCCGCAATGAGCTGTTGCGTACCCCCCTTTTTTAAGTTGTTGATTTTTAAGTCTTTCGCATTTCGCCCTATAT
CTAGTTCTTTGGTGCCCAAAGAAGGGCACCCCTGCGGGGTTCCCCCACGCCTTCGGCGCGGCTCCCCCTCCGGCAAAAAGT
GGCCCCTCCGGGGCTTGTTGATCGACTGCGCGGCCTTCGGCCTTGCCCAAGGTGGCGCTGCCCCCTTGGAACCCCCGCACT
CGCCGCCGTGAGGCTCGGGGGGCAGGCGGGCGGGCTTCGCCCTTCGACTGCCCCCACTCGCATAGGCTTGGGTCGTTCCAG
GCGCGTCAAGGCCAAGCCGCTGCGCGGTCGCTGCGCGAGCCTTGACCCGCCTTCCACTTGGTGTCCAACCGGCAAGCGAAG
CGCGCAGGCCGCAGGCCGGAGGCTTTTCCCCAGAGAAAATTAAAAAAATTGATGGGGCAAGGCCGCAGGCCGCGCAGTTGG
AGCCGGTGGGTATGTGGTCGAAGGCTGGGTAGCCGGTGGGCAATCCCTGTGGTCAAGCTCGTGGGCAGGCGCAGCCTGTCC
ATCAGCTTGTCCAGCAGGGTTGTCCACGGGCCGAGCGAAGCGAGCCAGCCGGTGGCCGCTCGCGGCCATCGTCCACATATC
CACGGGCTGGCAAGGGAGCGCAGCGACCGCGCAGGGCGAAGCCCGGAGAGCAAGCCCGTAGGGGGGGCGCGCCCAGCTGTC
TAGGGCGGCGGATTTGTCCTACTCAGGAGAGCGTTCACCGACAAACAACAGATAAAACGAAAGGCCCAGTCTTTCGACTGA
GCCTTTCGTTTTATTTGATGCCT

Figure S2. Nucleotide sequence of pSEVA237*Pa*. Hybridation sequences of oligonucleotides used to clone P_a into pSEVA237 backbone are underlined and restriction enzyme sites are shown in bold.

TTAATTAAAGCGGATAACAATTTCACACAGGAGGCCGCCTAGGCCGCGGCCGCGC**GAATTC**CTA<u>GGCAGCACCATCCGCCC</u>
CCCGCCCGTCGGCACCGGCTCCGGGCACATAGGCATCCCCGCCCGGCGCACGCCCCGGATCAAACCCCAGCACATCACACG
CGAAGGCGATCCATGCATCCGGATCCGGAACATCGAGCCCGACATAGCCCAGACCGAGAATTTCCATGCACCTCTCCCAAA
AAACGCACTTTCGATACTATTGGTACTGTTATTAGGAGTCAAGAGACCCGGCCAGCAGATTGTGCCATGAACACAGGGCTG
GACAGACAGAAATTTTATAGGTACCGACAGTTTCGTTAATGGCA<u>ACTCCGGCGCGCAACC</u>**GTCGAC**CTGCAGGCATGCAAG
CTTAGGAGGAAAAACATATGAGTAAAGGAGAAGAACTTTTCACTGGAGTTGTCCCAATTCTTGTTGAATTAGATGGTGATG
TTAATGGGCACAAATTTTCTGTCAGTGGAGAGGGTGAAGGTGATGCAACATACGGAAAACTTACCCTTAAATTTATTTGCA
CTACTGGAAAACTACCTGTTCCATGGCCAACACTTGTCACTACTTTGACTTATGGTGTTCAATGCTTTTCAAGATACCCAG
ATCATATGAAACGGCATGACTTTTTCAAGAGTGCCATGCCCGAAGGTTATGTACAGGAAAGAACTATATTTTTCAAAGATG
ACGGGAACTACAAGACACGTGCTGAAGTCAAGTTTGAAGGTGATACCCTTGTTAATAGAATCGAGTTAAAAGGTATTGATT
TTAAAGAAGATGGAAACATTCTTGGACACAAATTGGAATACAACTATAACTCACACAATGTATACATCATGGCAGACAAAC
AAAAGAATGGAATCAAAGTTAACTTCAAAATTAGACACAACATTGAAGATGGAAGCGTTCAACTAGCAGACCATTATCAAC
AAAATACTCCAATTGGCGATGGCCCTGTCCTTTTACCAGACAACCATTACCTGTCCACACAATCTGCCCTTTCGAAAGATC
CCAACGAAAAGAGAGACCACATGGTCCTTCTTGAGTTTGTAACAGCTGCTGGGATTACACATGGCATGGATGAACTATACA
AATAGCATTCGTATGTCTGCTATTTCGCGTATAGAACTAGTCTTGGACTCCTGTTGATAGATCCAGTAATGACCTCAGAAC
TCCATCTGGATTTGTTCAGAACGCTCGGTTGCCGCCGGGCGTTTTTTATTGGTGAGAATCCAGGGGTCCCCAATAATTACG
ATTTAAATTTGTGTCTCAAAATCTCTGATGTTACATTGCACAAGATAAAAATATATCATCATGAACAATAAAACTGTCTGC
TTACATAAACAGTAATACAAGGGGTGTTATGAGCCATATTCAGCGTGAAACGAGCTGTAGCCGTCCGCGTCTGAACAGCAA
CATGGATGCGGATCTGTATGGCTATAAATGGGCGCGTGATAACGTGGGTCAGAGCGGCGCGACCATTTATCGTCTGTATGG
CAAACCGGATGCGCCGGAACTGTTTCTGAAACATGGCAAAGGCAGCGTGGCGAACGATGTGACCGATGAAATGGTGCGTCT
GAACTGGCTGACCGAATTTATGCCGCTGCCGACCATTAAACATTTTATTCGCACCCCGGATGATGCGTGGCTGCTGACCAC
CGCGATTCCGGGCAAAACCGCGTTTCAGGTGCTGGAAGAATATCCGGATAGCGGCGAAAACATTGTGGATGCGCTGGCCGT
GTTTCTGCGTCGTCTGCATAGCATTCCGGTGTGCAACTGCCCGTTTAACAGCGATCGTGTGTTTCGTCTGGCCCAGGCGCA
GAGCCGTATGAACAACGGCCTGGTGGATGCGAGCGATTTTGATGATGAACGTAACGGCTGGCCGGTGGAACAGGTGTGGAA
AGAAATGCATAAACTGCTGCCGTTTAGCCCGGATAGCGTGGTGACCCACGGCGATTTTAGCCTGGATAACCTGATTTTCGA
TGAAGGCAAACTGATTGGCTGCATTGATGTGGGCCGTGTGGGCATTGCGGATCGTTATCAGGATCTGGCCATTCTGTGGAA
CTGCCTGGGCGAATTTAGCCCGAGCCTGCAAAAACGTCTGTTTCAGAAATATGGCATTGATAATCCGGATATGAACAAACT
GCAATTTCATCTGATGCTGGATGAATTTTTCTAATAATTAATTGGACCGCGGTCCGCGCGTTGTCCTTTTCCGCTGCATAA
CCCTGCTTCGGGGTCATTATAGCGATTTTTTCGGTATATCCATCCTTTTTCGCACGATATACAGGATTTTGCCAAAGGGTT
CGTGTAGACTTTCCTTGGTGTATCCAACGGCGTCAGCCGGGCAGGATAGGTGAAGTAGGCCCACCCGCGAGCGGGTGTTCC
TTCTTCACTGTCCCTTATTCGCACCTGGCGGTGCTCAACGGGAATCCTGCTCTGCGAGGCTGGCCGTAGGCCGGCCCTACC
GGCGCGGCAGCGTTACCCGTGTCGGCGGCTCCAACGGCTCGCCATCGTCCAGAAAACACGGCTCATCGGGCATCGGCAGGC
GCTGCTGCCCGCGCCGTTCCCATTCCTCCGTTTCGGTCAAGGCTGGCAGGTCTGGTTCCATGCCCGGAATGCCGGGCTGGC
TGGGCGGCTCCTCGCCGGGGCCGGTCGGTAGTTGCTGCTCGCCCGGATACAGGGTCGGGATGCGGCGCAGGTCGCCATGCC
CCAACAGCGATTCGTCCTGGTCGTCGTGATCAACCACCACGGCGGCACTGAACACCGACAGGCGCAACTGGTCGCGGGGCT
GGCCCCACGCCACGCGGTCATTGACCACGTAGGCCGACACGGTGCCGGGGCCGTTGAGCTTCACGACGGAGATCCAGCGCT
CGGCCACCAAGTCCTTGACTGCGTATTGGACCGTCCGCAAAGAACGTCCGATGAGCTTGGAAAGTGTCTTCTGGCTGACCA
CCACGGCGTTCTGGTGGCCCATCTGCGCCACGAGGTGATGCAGCAGCATTGCCGCCGTGGGTTTCCTCGCAATAAGCCCGG
CCCACGCCTCATGCGCTTTGCGTTCCGTTTGCACCCAGTGACCGGGCTTGTTCTTGGCTTGAATGCCGATTTCTCTGGACT
GCGTGGCCATGCTTATCTCCATGCGGTAGGGGTGCCGCACGGTTGCGGCACCATGCGCAATCAGCTGCAACTTTTCGGCAG
CGCGACAACAATTATGCGTTGCGTAAAAGTGGCAGTCAATTACAGATTTTCTTTAACCTACGCAATGAGCTATTGCGGGGG
GTGCCGCAATGAGCTGTTGCGTACCCCCCTTTTTTAAGTTGTTGATTTTTAAGTCTTTCGCATTTCGCCCTATATCTAGTT
CTTTGGTGCCCAAAGAAGGGCACCCCTGCGGGGTTCCCCCACGCCTTCGGCGCGGCTCCCCCTCCGGCAAAAAGTGGCCCC
TCCGGGGCTTGTTGATCGACTGCGCGGCCTTCGGCCTTGCCCAAGGTGGCGCTGCCCCCTTGGAACCCCCGCACTCGCCGC
CGTGAGGCTCGGGGGGCAGGCGGGCGGGCTTCGCCCTTCGACTGCCCCCACTCGCATAGGCTTGGGTCGTTCCAGGCGCGT
CAAGGCCAAGCCGCTGCGCGGTCGCTGCGCGAGCCTTGACCCGCCTTCCACTTGGTGTCCAACCGGCAAGCGAAGCGCGCA
GGCCGCAGGCCGGAGGCTTTTCCCCAGAGAAAATTAAAAAAATTGATGGGGCAAGGCCGCAGGCCGCGCAGTTGGAGCCGG
TGGGTATGTGGTCGAAGGCTGGGTAGCCGGTGGGCAATCCCTGTGGTCAAGCTCGTGGGCAGGCGCAGCCTGTCCATCAGC
TTGTCCAGCAGGGTTGTCCACGGGCCGAGCGAAGCGAGCCAGCCGGTGGCCGCTCGCGGCCATCGTCCACATATCCACGGG
CTGGCAAGGGAGCGCAGCGACCGCGCAGGGCGAAGCCCGGAGAGCAAGCCCGTAGGGGGGGCGCGCCCAGCTGTCTAGGGC
GGCGGATTTGTCCTACTCAGGAGAGCGTTCACCGACAAACAACAGATAAAACGAAAGGCCCAGTCTTTCGACTGAGCCTTT
CGTTTTATTTGATGCCT

Figure S3. Nucleotide sequence of pSEVA237*Pb*. Hybridation sequences of oligonucleotides used to clone P_b into pSEV237 backbone are underlined and restriction enzyme sites are shown in bold.

TTAATTAAAGCGGATAACAATTTCACACAGGAGGCCGCCTAGGCCGCGGCCGCGC**GAATTC**CTAATCGTCGTAGATGGCGG
AAACCGCGGCGGGATTCTGTTCGCATGCCCTGACAGCGGAAGTAGGAAAGCGGTCGTCGCATAACGCCCGCACGGAGCGCT
GCAGCGCGTCCTGCTCTTCCGATATGCTGAGGTCCATCCCGCTCTTCTCCCATCCGGTTGCGCGCCGGAGTTGCCATTAAC
GAAACTGTCGGTACCTATAAAATTTCTGTCTGTCCAGCCCTGTGTTCATGGCACAATCTGCTGGCCGGGTCTCTTGACTCC
TAATAACAGTACCAATAGTATCGAAAGTGCGTTTTTT**GTCGA***C*CTGCAGGCATGCAAGCTTAGGAGGAAAAACATATGAGT
AAAGGAGAAGAACTTTTCACTGGAGTTGTCCCAATTCTTGTTGAATTAGATGGTGATGTTAATGGGCACAAATTTTCTGTC
AGTGGAGAGGGTGAAGGTGATGCAACATACGGAAAACTTACCCTTAAATTTATTTGCACTACTGGAAAACTACCTGTTCCA
TGGCCAACACTTGTCACTACTTTGACTTATGGTGTTCAATGCTTTTCAAGATACCCAGATCATATGAAACGGCATGACTTT
TTCAAGAGTGCCATGCCCGAAGGTTATGTACAGGAAAGAACTATATTTTTCAAAGATGACGGGAACTACAAGACACGTGCT
GAAGTCAAGTTTGAAGGTGATACCCTTGTTAATAGAATCGAGTTAAAAGGTATTGATTTTAAAGAAGATGGAAACATTCTT
GGACACAAATTGGAATACAACTATAACTCACACAATGTATACATCATGGCAGACAAACAAAAGAATGGAATCAAAGTTAAC
TTCAAAATTAGACACAACATTGAAGATGGAAGCGTTCAACTAGCAGACCATTATCAACAAAATACTCCAATTGGCGATGGC
CCTGTCCTTTTACCAGACAACCATTACCTGTCCACACAATCTGCCCTTTCGAAAGATCCCAACGAAAAGAGAGACCACATG
GTCCTTCTTGAGTTTGTAACAGCTGCTGGGATTACACATGGCATGGATGAACTATACAAATAGCATTCGTATGTCTGCTAT
TTCGCGTATAGAACTAGTCTTGGACTCCTGTTGATAGATCCAGTAATGACCTCAGAACTCCATCTGGATTTGTTCAGAACG
CTCGGTTGCCGCCGGGCGTTTTTTATTGGTGAGAATCCAGGGGTCCCCAATAATTACGATTTAAATTTGTGTCTCAAAATC
TCTGATGTTACATTGCACAAGATAAAAATATATCATCATGAACAATAAAACTGTCTGCTTACATAAACAGTAATACAAGGG
GTGTTATGAGCCATATTCAGCGTGAAACGAGCTGTAGCCGTCCGCGTCTGAACAGCAACATGGATGCGGATCTGTATGGCT
ATAAATGGGCGCGTGATAACGTGGGTCAGAGCGGCGCGACCATTTATCGTCTGTATGGCAAACCGGATGCGCCGGAACTGT
TTCTGAAACATGGCAAAGGCAGCGTGGCGAACGATGTGACCGATGAAATGGTGCGTCTGAACTGGCTGACCGAATTTATGC
CGCTGCCGACCATTAAACATTTTATTCGCACCCCGGATGATGCGTGGCTGCTGACCACCGCGATTCCGGGCAAAACCGCGT
TTCAGGTGCTGGAAGAATATCCGGATAGCGGCGAAAACATTGTGGATGCGCTGGCCGTGTTTCTGCGTCGTCTGCATAGCA
TTCCGGTGTGCAACTGCCCGTTTAACAGCGATCGTGTGTTTCGTCTGGCCCAGGCGCAGAGCCGTATGAACAACGGCCTGG
TGGATGCGAGCGATTTTGATGATGAACGTAACGGCTGGCCGGTGGAACAGGTGTGGAAAGAAATGCATAAACTGCTGCCGT
TTAGCCCGGATAGCGTGGTGACCCACGGCGATTTTAGCCTGGATAACCTGATTTTCGATGAAGGCAAACTGATTGGCTGCA
TTGATGTGGGCCGTGTGGGCATTGCGGATCGTTATCAGGATCTGGCCATTCTGTGGAACTGCCTGGGCGAATTTAGCCCGA
GCCTGCAAAAACGTCTGTTTCAGAAATATGGCATTGATAATCCGGATATGAACAAACTGCAATTTCATCTGATGCTGGATG
AATTTTTCTAATAATTAATTGGACCGCGGTCCGCGCGTTGTCCTTTTCCGCTGCATAACCCTGCTTCGGGGTCATTATAGC
GATTTTTTCGGTATATCCATCCTTTTTCGCACGATATACAGGATTTTGCCAAAGGGTTCGTGTAGACTTTCCTTGGTGTAT
CCAACGGCGTCAGCCGGGCAGGATAGGTGAAGTAGGCCCACCCGCGAGCGGGTGTTCCTTCTTCACTGTCCCTTATTCGCA
CCTGGCGGTGCTCAACGGGAATCCTGCTCTGCGAGGCTGGCCGTAGGCCGGCCCTACCGGCGCGGCAGCGTTACCCGTGTC
GGCGGCTCCAACGGCTCGCCATCGTCCAGAAAACACGGCTCATCGGGCATCGGCAGGCGCTGCTGCCCGCGCCGTTCCCAT
TCCTCCGTTTCGGTCAAGGCTGGCAGGTCTGGTTCCATGCCCGGAATGCCGGGCTGGCTGGGCGGCTCCTCGCCGGGGCCG
GTCGGTAGTTGCTGCTCGCCCGGATACAGGGTCGGGATGCGGCGCAGGTCGCCATGCCCCAACAGCGATTCGTCCTGGTCG
TCGTGATCAACCACCACGGCGGCACTGAACACCGACAGGCGCAACTGGTCGCGGGGCTGGCCCCACGCCACGCGGTCATTG
ACCACGTAGGCCGACACGGTGCCGGGGCCGTTGAGCTTCACGACGGAGATCCAGCGCTCGGCCACCAAGTCCTTGACTGCG
TATTGGACCGTCCGCAAAGAACGTCCGATGAGCTTGGAAAGTGTCTTCTGGCTGACCACCACGGCGTTCTGGTGGCCCATC
TGCGCCACGAGGTGATGCAGCAGCATTGCCGCCGTGGGTTTCCTCGCAATAAGCCCGGCCCACGCCTCATGCGCTTTGCGT
TCCGTTTGCACCCAGTGACCGGGCTTGTTCTTGGCTTGAATGCCGATTTCTCTGGACTGCGTGGCCATGCTTATCTCCATG
CGGTAGGGGTGCCGCACGGTTGCGGCACCATGCGCAATCAGCTGCAACTTTTCGGCAGCGCGACAACAATTATGCGTTGCG
TAAAAGTGGCAGTCAATTACAGATTTTCTTTAACCTACGCAATGAGCTATTGCGGGGGGTGCCGCAATGAGCTGTTGCGTA
CCCCCCTTTTTTAAGTTGTTGATTTTTAAGTCTTTCGCATTTCGCCCTATATCTAGTTCTTTGGTGCCCAAAGAAGGGCAC
CCCTGCGGGGTTCCCCCACGCCTTCGGCGCGGCTCCCCCTCCGGCAAAAAGTGGCCCCTCCGGGGCTTGTTGATCGACTGC
GCGGCCTTCGGCCTTGCCCAAGGTGGCGCTGCCCCCTTGGAACCCCCGCACTCGCCGCCGTGAGGCTCGGGGGGCAGGCGG
GCGGGCTTCGCCCTTCGACTGCCCCCACTCGCATAGGCTTGGGTCGTTCCAGGCGCGTCAAGGCCAAGCCGCTGCGCGGTC
GCTGCGCGAGCCTTGACCCGCCTTCCACTTGGTGTCCAACCGGCAAGCGAAGCGCGCAGGCCGCAGGCCGGAGGCTTTTCC
CCAGAGAAAATTAAAAAAATTGATGGGGCAAGGCCGCAGGCCGCGCAGTTGGAGCCGGTGGGTATGTGGTCGAAGGCTGGG
TAGCCGGTGGGCAATCCCTGTGGTCAAGCTCGTGGGCAGGCGCAGCCTGTCCATCAGCTTGTCCAGCAGGGTTGTCCACGG
GCCGAGCGAAGCGAGCCAGCCGGTGGCCGCTCGCGGCCATCGTCCACATATCCACGGGCTGGCAAGGGAGCGCAGCGACCG
CGCAGGGCGAAGCCCGGAGAGCAAGCCCGTAGGGGGGGCGCGCCCAGCTGTCTAGGGCGGCGGATTTGTCCTACTCAGGAG
AGCGTTCACCGACAAACAACAGATAAAACGAAAGGCCCAGTCTTTCGACTGAGCCTTTCGTTTTATTTGATGCCT

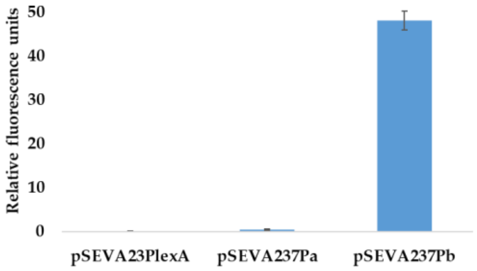

Figure S4. Fluorescence intensity of the *E. coli* DH10B (pSEVA23*PlexA*) as control, *E. coli* DH10B (pSEVA237*Pa*) and *E. coli* DH10B (pSEVA237*Pb*) strains carrying the transcriptional fusions of the P_a, P_b and P_t promoter regions, respectively. The values correspond to the mean of three independent biological replicas (n=3) and error bars correspond to SD.
